# In Silico Models Using Simple Molecular Descriptors Predict Placental and Breast Milk Transfer of Cannabinoids from *Cannabis sativa*

**DOI:** 10.3390/ijms27146446

**Published:** 2026-07-20

**Authors:** Anna W. Sobańska, Adam Hekner, Kinga Maciejek, Andrzej M. Sobański

**Affiliations:** 1 Department of Analytical Chemistry, Medical University of Lodz, 90-151 Łódź, Poland; adam.hekner@umed.lodz.pl (A.H.); kinga.maciejek@umed.lodz.pl (K.M.); 2 Faculty of Chemistry, University of Lodz, 91-403 Łódź, Poland; andrzej.sobanski@inwat-energo.pl

**Keywords:** *Cannabis sativa* cannabinoids, OC-PLS classification, placental permeability, QSAR models, secretion into breast milk, SIMCA classification

## Abstract

Despite an increasing interest in the pharmacology of cannabinoids from *Cannabis sativa*, little is known to date about their ability to cross the placenta and to be secreted into breast milk, and in this study, we sought to fill this gap. In total, 126 phytocannabinoids previously detected in *Cannabis sativa* were investigated for their transplacental transfer and secretion into breast milk. Placental transport was predicted using novel multiple linear regression (MLR), artificial neural network (ANN), boosted trees (BT), and support vector regression (SVR) models, based on a reference set of 84 compounds for which the placental clearance index (***CI***) relative to antipyrine is known. Secretion into breast milk was predicted using newly developed classification models based on soft independent modeling of class analogies (SIMCA) and One-Class Partial Least Squares (OC-PLS) algorithms. Analysis of the Q vs. Hotelling’s T^2^ plot for the cannabinoids indicated that they are similar in their physicochemical properties to compounds empirically demonstrated to enter breast milk (“in-class”); only 7 of 126 compounds were borderline (with elevated Q but not T^2^); no compounds were classified as “out-of-class”. The mean predicted ***CI*** values for phytocannabinoids investigated in this study ranged from 0.4 to 0.85. It was concluded that all the cannabinoids in the studied group might cross the placenta (although their passage might be expected to be more difficult than that of antipyrine) and enter breast milk. These results should support informed risk assessment and prioritization of cannabinoids for future experimental testing.

## 1. Introduction

Cannabinoids are the terpenophenolic actives of *Cannabis sativa* (hemp), used for over 4000 years as a recreational drug for its mind-altering effects [1]. The hemp plant contains over 500 ingredients, not all of which have been identified [2,3]. The main psychoactive (and psychotoxic) constituent of hemp is Δ^9^-tetrahydrocannabinol (Δ^9^-THC), naturally occurring in plants as the non-psychoactive tetrahydrocannabinolic acid (Δ^9^-THCA), which is decarboxylated to Δ^9^-THC upon heating [4]. THC consumption in excessive amounts can lead to mental, gastrointestinal, and cardiovascular problems in the general population, and prolonged use is linked to cognitive deficits, increased risk of psychosis/schizophrenia, mood and anxiety disorders, and suicidal behaviors in adolescents and young adults [5,6,7,8,9,10,11]. For that reason, the legal status of *Cannabis* preparations varies among countries, with the level of THC being the main factor distinguishing legal from illegal products [12]. *Cannabis* shares some cognitive and psychomotor effects with alcohol, yet it is perceived as more hazardous [13].

Other main phytocannabinoids (including cannabidiol, CBD, and cannabinol, CBN) are generally considered non-psychoactive [5,6,7,14], although they do have certain effects on mood (for example, CBD has anxiolytic properties) [15]. *Cannabis* actives, including THC and CBD, have antioxidant and anti-inflammatory properties [14,16] and may become useful in treating conditions such as chronic pain, multiple sclerosis, epilepsy, anxiety, cancer, and Parkinson’s disease [16,17,18,19]. Specifically, CBD is considered a promising antipsychotic, anti-inflammatory, and antiepileptic drug, and Δ^9^-tetrahydrocannabivarin (THCV) may find its place in the treatment of type 2 diabetes [20]. Cannabinoids interact with two G protein-coupled receptors (GPCRs)-cannabinoid receptors CB1 and CB2 [1]. CB1 receptors are found primarily in brain regions responsible for memory, sleep, emotions, coordination of movements, and body posture, including the cortex, forebrain, limbic system (hippocampus, hypothalamus, amygdala), striatum (basal ganglia, nucleus accumbens, substantia nigra, globus pallidus), and cerebellum [2]. CB2 receptors are primarily located in immune tissues, and they are involved in modulating immune responses [21]. None of the known cannabinoids is entirely CB1- or CB2-specific, and they can also interact with other targets, such as TRPV1, GPR55, and GPR119 [22]. Through these multiple molecular targets, cannabinoids can affect not only the central nervous and immune systems, but also reproductive and developmental processes.

Cannabis use can affect male and female fertility and has been found to interfere with reproductive hormones, menstrual cycles, and semen parameters. In men, it is believed to cause erectile dysfunction, abnormal spermatogenesis, and testicular atrophy. In females, the effects of cannabis exposure during reproductive age may include infertility (e.g., decreased ovarian reserve), abnormal embryo implantation, and development [23,24]. Some cannabinoids from *Cannabis sativa*, mainly THC and CBD, have been found to cross the placenta [25,26], although transplacental transfer is restricted to some degree. For example, due to albumin binding, injected CBD reaches concentrations in fetal blood plasma that are about 50% of those in maternal plasma [27]. THC appears to be actively effluxed by the human placenta, and the metabolites 11-OH-THC and COOH-THC passively diffuse across the placenta [28,29]. The placenta acts as a depot compartment for CBD, thereby slowing its distribution to the fetus [30]. A study on animal models demonstrated that THC and CBD accumulate in the maternal plasma after repeated use [31]. THC and its metabolites cross the placenta and are found in the fetal brain, at concentrations relative to those in the mother’s blood depending on the route of administration (inhalation vs. subcutaneous injection) [32]; CBD can also cross the fetal and neonatal blood–brain barrier [27].

Cannabis does not act as a classical teratogen and is not associated with morphological abnormalities at birth. However, neonates exposed to cannabis in utero had significantly lower total brain volume than those without a history of prenatal exposure [33]. Cannabis-affected pregnancies exhibited symptoms of restricted prenatal growth, linked to changes in placental vasculature and function [34,35]. Due to its interactions with cannabinoid receptors (THC is known to disrupt the endocannabinoid system’s homeostasis [36]), THC may affect the development and function of the pancreas, leading to metabolic issues (e.g., type II diabetes) later in life [24]. Global intelligence scores in children exposed to cannabis prenatally are unaffected; however, some negative impact on cognitive functions such as attention, inhibitory control, and planning has been reported, along with higher levels of depression and anxiety during adolescence [23,37]. THC-exposed pregnancies showed symptoms of placental insufficiency, such as decreased amniotic fluid volume, placental perfusion, and fetal oxygen availability [38].

Cannabis use among breastfeeding women is rising, and multiple studies now show that THC and its metabolites are secreted into human breast milk [39,40,41,42,43]. Carboxy-THC, 11-hydroxy-THC, CBD, and CBN were also detected in the milk of breastfeeding cannabis users [44,45]. CBD enters breast milk at concentrations that depend on the maternal route of administration [46]. Maternal exposure to THC and CBD has been reported to alter breast milk composition (lipid, protein, and lactose concentrations), leading to higher protein and lower fat levels [44,47,48]. It is speculated that interactions between exogenous cannabinoids and the endocannabinoid system might modify the release of hormones that regulate milk secretion, such as prolactin and dopamine [49]. The variety of known and suspected outcomes of marijuana use during lactation, along with a high degree of uncertainty about its possible effects, supports the notion that mothers should refrain from recreational cannabis use during lactation. If marijuana is used to treat maternal health conditions during this period, other therapeutic options should be considered [50].

The pharmacology of less-abundant phytocannabinoids is understudied compared with that of the main compounds from this family, THC and CBD, and their metabolites, but this situation is likely to change. For example, 8 cannabinoids: ∆^9^-THC, ∆^9^-tetrahydrocannabinolic acid (∆^9^THCA), ∆^9^-tetrahydrocannabivarin (THCV), CBD, cannabidiolic acid (CBDA), cannabidivarin (CBDV), cannabigerol (CBG), and cannabichromene (CBC) were assessed for receptor affinity, ability to inhibit cAMP accumulation, β-arrestin2 recruitment, receptor selectivity, and ligand bias in cell culture; and for cataleptic, hypothermic, anti-nociceptive, hypolocomotive, and anxiolytic effects in mice [51]. However, little is known to date about the ability of minor cannabinoids from *Cannabis sativa* to cross the placenta and be secreted into breast milk. This lack of empirical data complicates toxicological risk assessment and supports the use of predictive approaches as an initial screening step.

In this study, we propose in silico models of placental transfer and secretion into human milk, built on experimental reference data and simple, readily obtainable, and mechanistically interpretable molecular descriptors. These models should enable a preliminary assessment of clinically and toxicologically relevant endpoints across a broad set of cannabinoids derived from *Cannabis sativa*. Our hypothesis is that such descriptor-based in silico models can capture key determinants of maternal–fetal and maternal–infant exposures beyond lipophilicity, and thus offer meaningful predictions for cannabinoids for which no empirical human data are yet available. In this sense, the present study contributes to filling the current data gap on cannabinoid safety in pregnancy and lactation and may support more informed risk assessment and the rational design of future investigations, including more resource-intensive in vitro experiments and animal models.

## 2. Results

### 2.1. The Placental Permeability of Cannabinoids

The placental clearance (***CI***) index is a dimensionless ratio that quantifies how efficiently a compound crosses the human placenta in ex vivo perfusion experiments by comparing its placental clearance to that of a reference compound that crosses the placenta freely (usually antipyrine). The experimental determination of ***CI*** is not possible in most cases for obvious ethical reasons, which is why in silico methods based on established experimental data are being developed.

**Figure 1:**
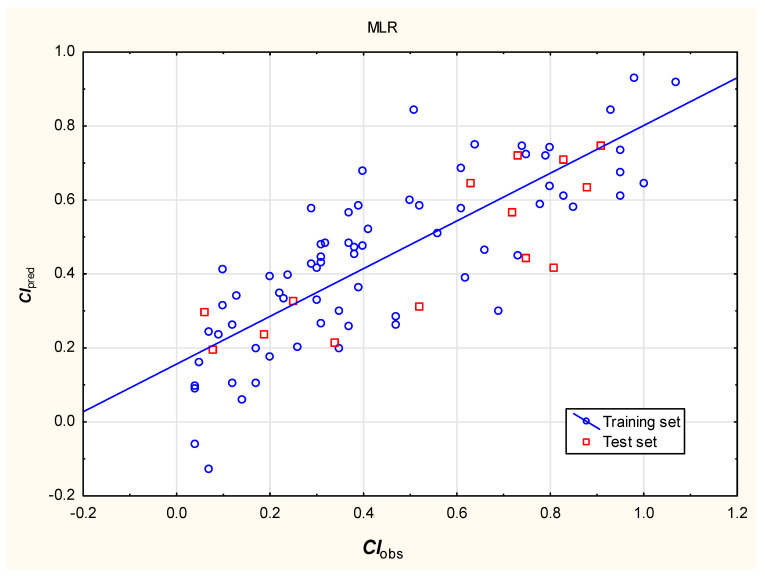
Predicted vs. observed—MLR model of ***CI***, Equation (1).

In this study, the ***CI*** of 126 cannabinoids was initially predicted using a novel multiple linear regression (MLR) equation (1), developed with easily interpretable molecular descriptors selected via stepwise (backward) regression (Figure 1).

***CI*** = 2.204 (±0.222) − 0.0157 (±0.0060) ***nRot*** − 0.000684 (±0.000307) ***mp*** + 0.278 (±0.050) ***caco2*** − 0.194 (±0.073) ***PAMPA*** (1)

(n = 70, R^2^ = 0.686, R^2^_adj._ = 0.645, Q^2^ = 0.584, RMSE_pred_ = 0.192, F = 29.5, *p* < 0.0001)

**Figure 2:**
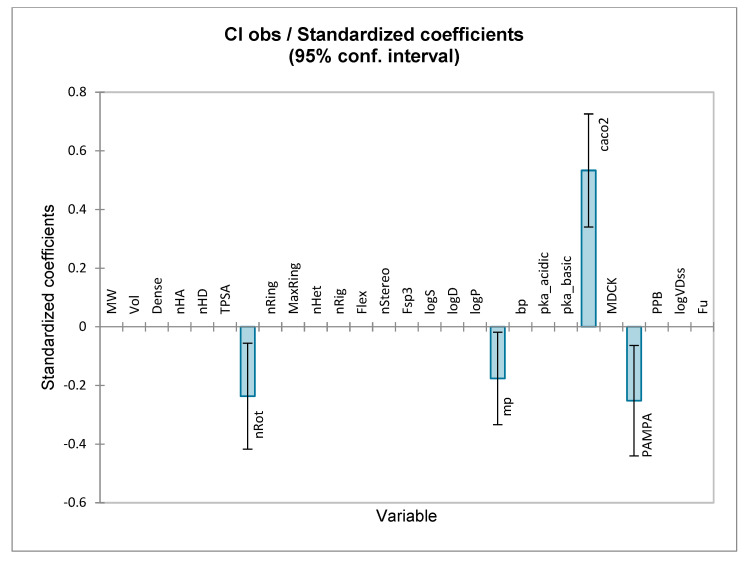
Standardized coefficients of Equation (1).

Equation (1) is a model of moderate quality; however, it points to one molecular property that is positively correlated with ***CI*** (Caco-2 permeability, ***caco2***), and to descriptors that are inversely correlated with ***CI*** (the count of rotatable bonds, ***nRot***; melting point, ***mp***; Parallel Artificial Membrane Permeability Assay, ***PAMPA***) (Figure 2). Melting point is not a common descriptor in QSAR analysis; however, it encodes several molecular properties related mainly to the molecule’s symmetry, eccentricity, chirality, flexibility, and hydrogen bonding–some of which also determine the ability of compounds to cross biological barriers [52,53]. Interestingly, Caco-2 and PAMPA permeabilities appear to exert opposite influences on ***CI***. However, both assays model different permeability mechanisms. Caco-2 permeability reflects passive diffusion, active uptake, efflux, and paracellular pathways, whereas PAMPA measures only passive transmembrane diffusion, excluding active transport, efflux, and paracellular routes, and is related to lipophilicity, ionization/charge distribution, polarity, hydrogen bonding pattern and shielding, size/shape, and specific interactions with lipids [54,55,56,57]. The ***PAMPA*** descriptor used in this study is, in fact, the probability that a compound is highly PAMPA-permeable; it has been found to be inversely correlated with compounds’ ability to bioconcentrate in aquatic organisms, their mobility in soil, and their permeability across the blood–brain barrier [58,59].

**Figure 3:**
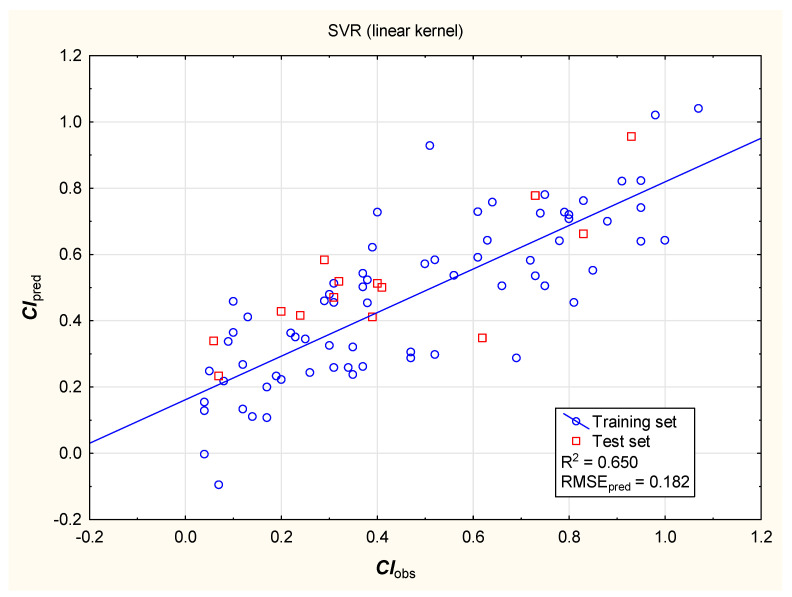
Predicted vs. observed—SVR model of ***CI***.

**Figure 4:**
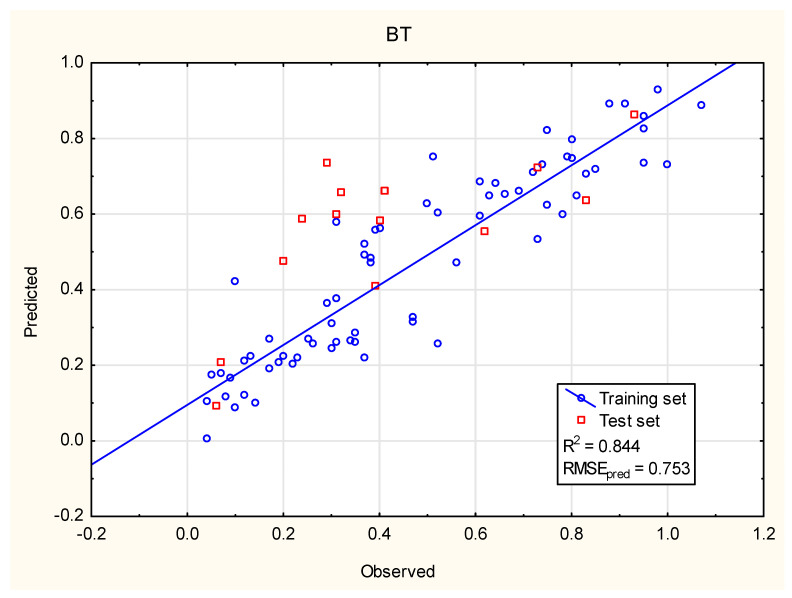
Predicted vs. observed—BT model of ***CI***.

**Figure 5:**
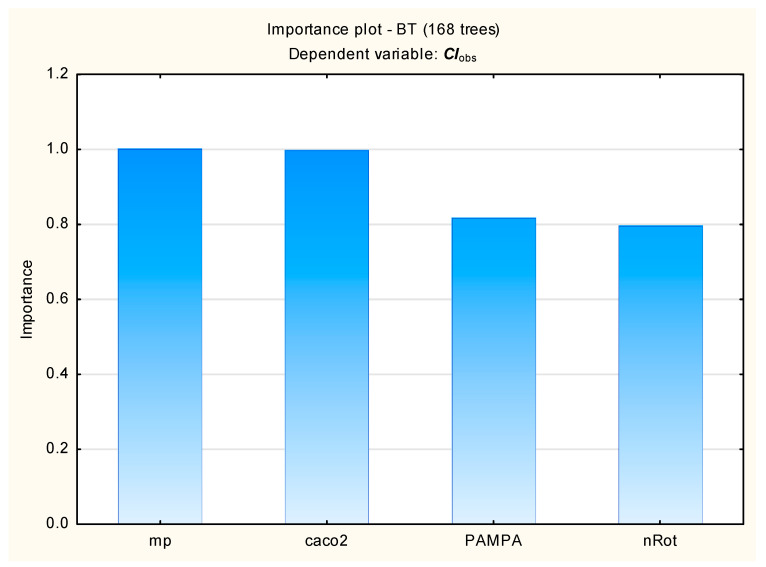
Importance of descriptors used in the Boosted Tree model of ***CI***.

**Figure 6:**
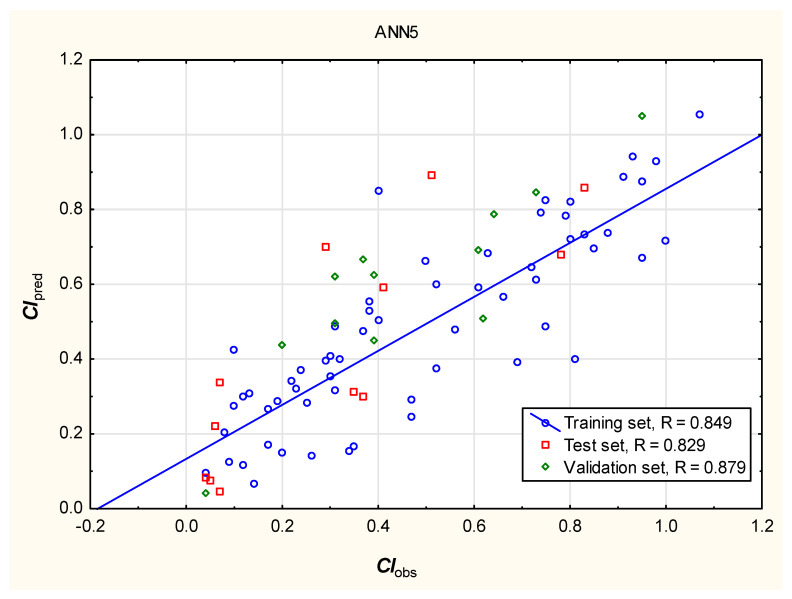
Predicted vs. observed—ANN model of ***CI***.

**Figure 7:**
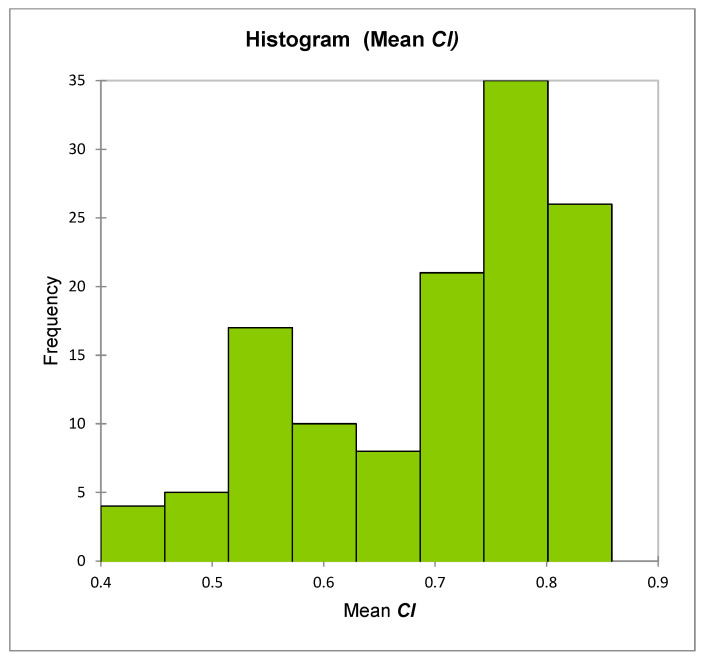
Distribution of mean predicted ***CI*** values for cannabinoids.

The same set of independent variables has been used to construct Support Vector Regression (SVR, Figure 3), Boosted Tree (BT, Figure 4 and Figure 5), and Artificial Neural Network (ANN, Figure 6) models, and the ***CI*** predictions are provided in the Supplementary Materials. Based on Figure 7, no compounds in the studied group have ***CI*** values indicating highly restricted transplacental passage.

**Figure 8:**
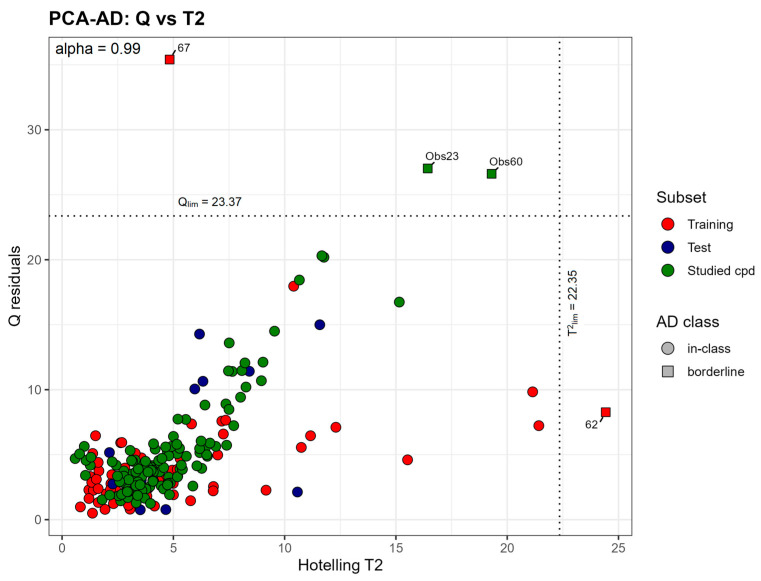
AD study of the ***CI*** models—reference compounds and cannabinoids in the Q-T^2^ coordinate system.

The applicability domain (AD) of the ***CI*** models developed in this study was assessed using Principal Component Analysis (PCA). Hotelling’s T^2^ (the score distance, expressed as the squared Mahalanobis distance from the origin of the score subspace) and Q (the orthogonal distance, expressed as the sum of squared residuals) [60,61] were calculated for the reference compounds, and plotted in the Q vs. Hotelling T^2^ coordinate system. The studied cannabinoids were superimposed on this plot (Figure 8). It was found that only two cannabinoids (Obs23 and Obs60) are borderline, and no compounds are typical outliers, thereby justifying the use of models based on the reference set proposed by Giaginis [62] for the compounds investigated in this study.

### 2.2. One-Class Models of Cannabinoids’ Ability to Enter Breast Milk

The secretion of xenobiotics into breast milk is an important route of unintentional infant exposure. Some cannabinoids from Cannabis sativa are known to enter mother’s milk; however, to the best of our knowledge, no systematic study has been conducted in this area. In this study, we sought to qualitatively evaluate the risk of cannabinoids’ secretion into breast milk, based on their similarities and differences relative to compounds whose secretion into breast milk has been experimentally established. At this stage, the decision was made to use a set of reference compounds with high affinity for human breast milk provided by Vijayaraghavan et al. [63] and to employ a one-class modeling strategy, in which the classification problem is addressed by examining instances of only one class, usually the class of interest [60,64]. The reference set selected for this study encompasses mainly pesticides and other environmental and food contaminants detected in breast milk that fall outside the typical chemical space considered in pharmacological studies (Supplementary Materials); the reference compounds have, on average, 0.87 violations of Lipinski’s Ro5 [65], mostly caused by their relatively high lipophilicity. Many of them are expected to exhibit poor oral availability and, in this respect, are similar to the studied group.

Hence, the ability of 126 cannabinoids to enter mother’s milk was qualitatively assessed using Soft Independent Modeling of Class Analogies (SIMCA). SIMCA is a classification method in which each class is modeled separately using a principal component analysis (PCA) model built from reference compounds of that class. A new sample is assigned to a class if it falls within the corresponding PCA model’s confidence limits [66,67,68,69]. It is called “soft” because each compound can be assigned to one class, multiple classes, or no class at all. It has been developed primarily for process control and identification, since in such situations the desired response is whether an object meets the requirements of a given class [70]. The compounds are analyzed in terms of their distances from the reference group using two measures: Hotelling’s T^2^ and Q [60,61]. The compounds with both T^2^ and Q below the respective thresholds are classified as “in-class”, those exceeding one threshold as “borderline”, and those exceeding both thresholds as “out-of-class” [71].

In this study, a reference set of 202 compounds for which the ability to enter breast milk has been demonstrated experimentally was randomly divided into two sets: a training set (n = 160) and a test set (external validation, n = 42). The number of PCs (K) was selected via 5-fold cross-validation on the training set, comparing candidate models with K = 1–10 PCs and selecting the simplest model that yielded a false-negative (FN) rate of 0. Hence, a model based on two PCs (K = 2) was constructed for the training set; 42 compounds from the test set were assessed using the model, and the results were visualized in the Q-T^2^ coordinate system. It was noted that 6 of 202 reference compounds, whose ability to enter breast milk has been experimentally determined, were classified as “borderline”, and no compounds were classified as false negatives, which demonstrates the satisfactory reliability of the SIMCA model.

**Figure 9:**
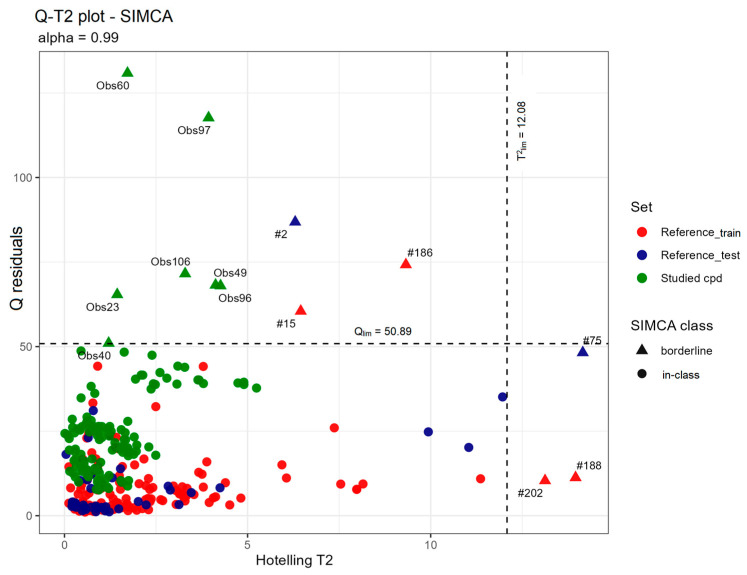
Q vs. T^2^ plot of the reference and studied compounds.

**Figure 10:**
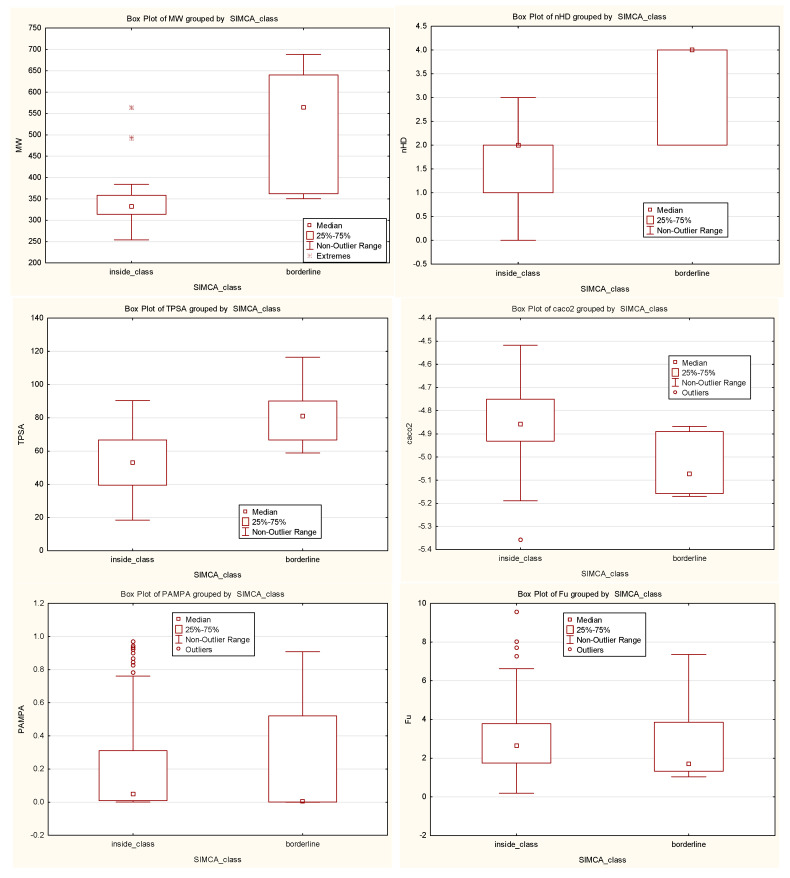
Comparison of selected descriptors for compounds classified as “in-class” and “borderline”.

Analysis of the Q vs. T^2^ plot for the cannabinoids (Figure 9) indicated that they might readily enter breast milk (“in-class”); only 7 of 126 compounds are borderline (with elevated Q but not T^2^); no compounds were classified as “out-of-class”. The molecules labeled “borderline” are typically bulkier, more polar, and more prone to H-bonding than those “in-class”; they differ in Caco-2 permeability and are similar in plasma-protein binding (similar Fu) and in membrane permeability by passive diffusion (PAMPA) (Figure 10). However, the “borderline” designation does not imply that these compounds cannot enter breast milk; it merely indicates that they are atypical and, hence, their passage into breast milk should be further investigated.

**Figure 11:**
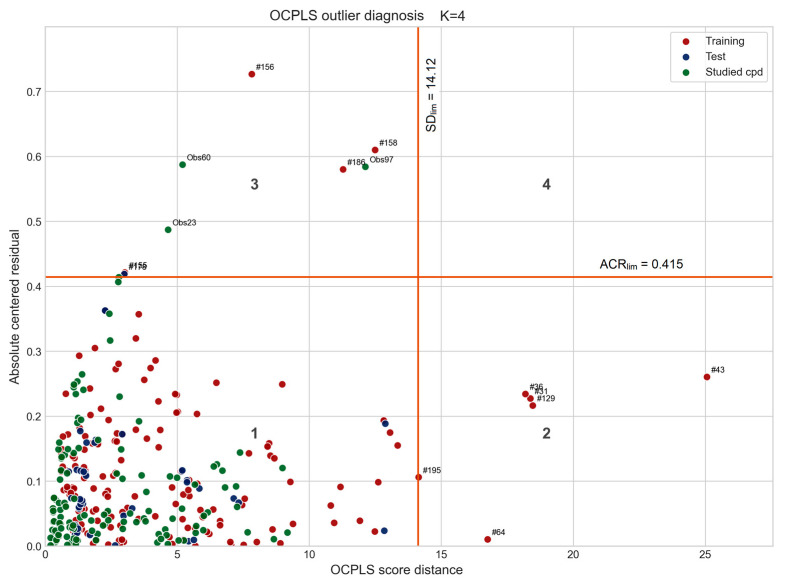
An OC-PLS outlier diagnostic plot based on the absolute values of centered model residuals of the response variable and the OC-PLS scores of primary latent variables. Region 1—regular points; Region 2—good leverage points; Region 3—class outliers; Region 4—bad leverage points.

The cannabinoids from *Cannabis sativa* were also evaluated for their ability to enter breast milk using the One-Class Partial Least Squares (OC-PLS) methodology [72]. In the OC-PLS model (Figure 11), the majority of training, test, and studied compounds were located in region 1, corresponding to regular points accepted by the model. Only a limited number of compounds from the reference and studied groups appeared in regions 2 and 3, whereas region 4 remained empty.

Compounds in Region 2 can be interpreted as good leverage points, i.e., points with relatively large score distances but low response residuals, whereas objects in Region 3 correspond to class outliers characterized by small score distances but elevated ACR values. It is worth stressing at this point that the absence of objects in Region 4 was consistent with the behavior previously observed in the SIMCA model, in which no compound exceeded both decision thresholds.

All cannabinoids assigned by OC-PLS to Regions 2 and 3 (Obs23, Obs60, and Obs97) were previously identified as “borderline” by the SIMCA algorithm. This qualitative agreement suggests that both one-class approaches capture a similar underlying structure of the data, despite relying on different model spaces and distance definitions.

**Figure 12:**
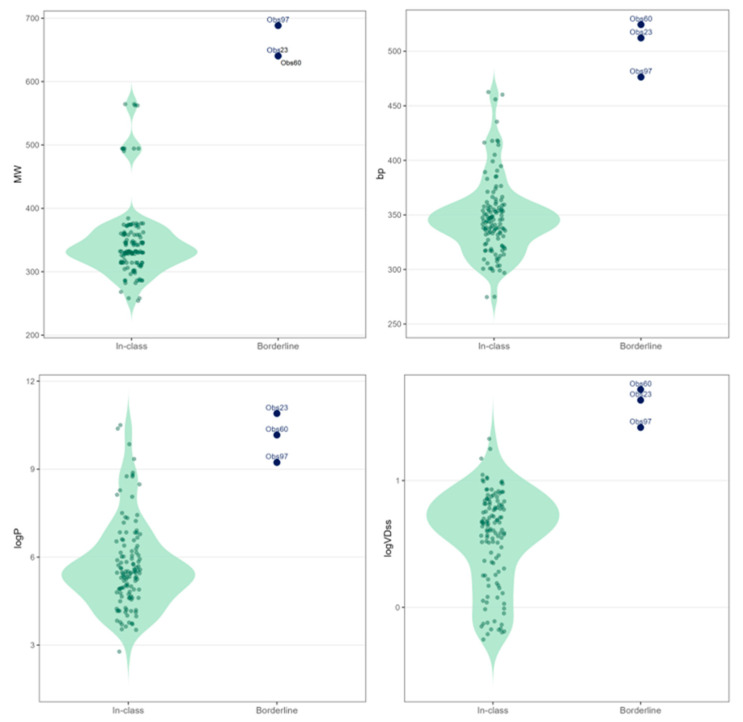
Key differences between Obs23, Obs60 and Obs97 and other cannabinoids.

**Figure 13:**
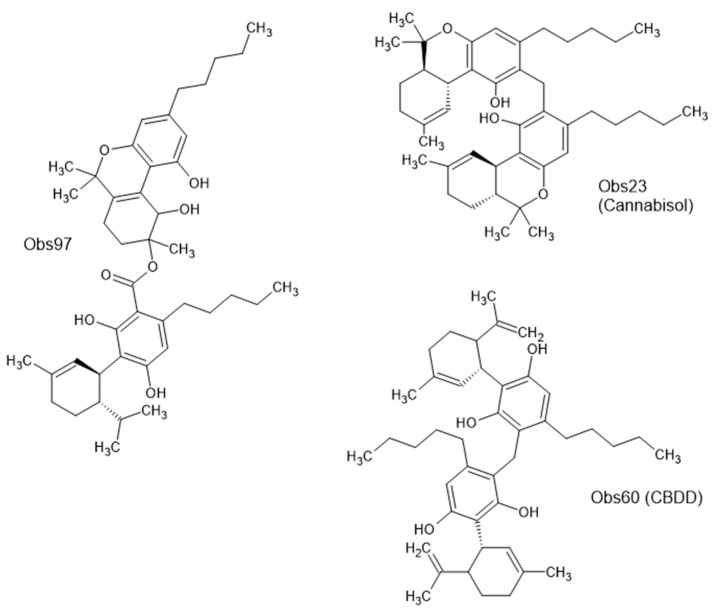
Structures of the most atypical cannabinoids in the context of secretion into breast milk.

Cannabinoids denoted as Obs23, Obs60, and Obs97 differ from other compounds in this family in some respects (Figure 12), particularly in molecular weight and predicted boiling point. It appears that two of them are dimeric structures (Obs23–cannabisol and Obs60–CBDD). The third one, Obs97, is a bulky CBDA-C5 9-OH-CBT-C5 ester (Figure 13).

## 3. Discussion

According to our predictions, all cannabinoids investigated in this study might cross the placenta, although their transplacental passage may be somewhat restricted. No compounds in the studied group appear to have a placental ***CI*** comparable to or exceeding that of antipyrine (***CI*** ≥ 1), a well-established reference compound that crosses the placenta freely, and no compounds have ***CI*** values below ca. 0.3–0.4, which would imply severely restricted passage. The ***CI*** values calculated for some cannabinoids that have recently attracted special attention in the context of other biological properties [51] suggest that they might cross the placenta, with THC, THCV, CBD, CBDV, CBC, and CBG among the compounds with the highest predicted ***CI*** values in the entire group of 126 cases (Table 1). The results for THC and CBD are consistent with existing reports on the transplacental transfer of these compounds [25,26].

Similarly, all cannabinoids might enter human breast milk (as previously reported for THC, CBD, and metabolites not investigated in this study, including 11-OH-THC and THC-COOH [40,41,44,45]). Some particularly bulky and lipophilic compounds from the studied group were classified as “borderline” by one or both of the one-class classification models developed in this study; however, although atypical, they are not sufficiently distant from the “in-class” compounds to warrant their safety during lactation.

The models developed herein have some limitations:

- Transplacental transfer: Models reported in this study do not account for cannabinoid metabolism in the human placenta and for possible excretion from the placenta and the fetal compartment. Future investigations should include these considerations.
- Secretion into breast milk: Models developed in this study are based on the similarity or dissimilarity of physicochemical properties between cannabinoids and the reference compounds detected in real breast milk samples. The reference group used in this study was derived from analyses of human milk samples for the presence of chemicals that are expected to be an issue for the environment and public health, rather than from a dataset specifically designed to cover the full range of physicochemical properties of substances to which a pregnant or lactating woman may be exposed.

However, using one-class models to predict the ability of cannabinoids to enter mother’s milk has the practical advantage that the results are not obscured by methodological differences in the quantitative reference data scattered across different literature sources, such as sampling schemes and analytical methods.

## 4. Materials and Methods

### 4.1. Compounds

Structures of 126 cannabinoids identified in *Cannabis sativa* were taken from Radwan et al. [3]. 202 compounds with a known high risk of secretion into human breast milk were taken from Vijayaraghavan et al. [63], and the reference compounds used to generate the QSAR models of the placental permeability were from Giaginis et al. [62]. SMILES strings for all compounds were retrieved from PubChem or generated in ACDLabs ChemSketch v.2023.1.2 (Supplementary Materials).

### 4.2. Descriptors

Molecular descriptors used in this study were calculated using ADMETLab3.0 software [73], with SMILES strings as input. The physicochemical descriptors used in this study are: molecular weight (***MW***); topological polar surface area (***TPSA***); count of hydrogen bond donors (***nHD***); count of rotatable bonds (***nRot***); count of non-carbon atoms (hydrogens included) (***nHet***); count of rigid bonds (***nRig***); ***nRot***/***nRig*** (***Flex***); logarithmic n-octanol/water partition coefficient (log***P***); fraction of sp^3^-hybridized carbon atoms (***Fsp3***); boiling point (***bp***); melting point (***mp***); acidic dissociation constant (***pka_acidic***); basic dissociation constant (***pka_basic***). ADMET properties considered in the study are: calculated permeabilities (***caco2***; ***MDCK***; ***PAMPA***), where ***caco2*** and ***MDCK*** are quantitative permeability data, and ***PAMPA*** is a probability that a compound is PAMPA permeable; volume of distribution at steady state (***VDss***); and fraction unbound in plasma, % (***Fu***). Descriptor values for the reference compounds and cannabinoids are provided in the Supplementary Materials, along with the correlations between the descriptors for the sets of reference data used to predict both endpoints (placental permeability and secretion into breast milk). The set of descriptors used in SIMCA and OC-PLS models was selected to avoid collinearity; pairs of descriptors with very high Pearson correlations (|r| ≥ 0.9) were identified, and one variable was removed as appropriate.

### 4.3. Statistical and Chemometric Analysis

#### 4.3.1. Multiple Linear Regression (MLR) Model of ***CI***

The MLR model was generated in XLSTAT 2025.1 from Lumivero, using descriptors calculated by ADMETLab3.0, in stepwise backwards mode (probability of removal: 0.1), with the tolerance level set at 0.1 (it is assumed that two descriptors are collinear if the tolerance value between them, calculated as (1 − R^2^), is <0.1 [74]). 70 of 84 reference compounds were randomly assigned to the training set, and the remaining 14 to the test set. The MLR model was validated using R^2^, R^2^_adj._, and Q^2^ metrics for the training set, and RMSE_pred_ (Root Mean Square Error of prediction) for the test set [75,76,77].

#### 4.3.2. Artificial Neural Network (ANN) Models of ***CI***

Multilayer Perceptron (MLP) artificial neural networks (ANNs) were generated using Statistica v.13.3 (regression, Automated Network Search—ANS module, 500 networks to train, 50 networks to retain), based on the same set of independent variables as in the MLR analysis. The neuron activation functions used in this study were selected from the following set: identity, logistic, hyperbolic tangent, and exponential. The networks were trained with the BFGS (Broyden–Fletcher–Goldfarb–Shanno) algorithm. The error function was the sum of squares (SOS). The ANN models were evaluated using correlation coefficients for the training, test, and validation sets. The importance of independent variables in the ANN models was evaluated using global sensitivity analysis (GSA) by computing the sum of squared residuals for the model without that variable relative to the full model. In GSA, an input variable is considered redundant if its score does not exceed 1. Detailed data on the five retained networks are provided in the Supplementary Materials.

#### 4.3.3. Support Vector Regression (SVR) Model of ***CI***

The Support Vector Regression model was generated in XLSTAT 2025.1 using the same set of independent variables as in the MLR analysis and the same split of the reference compounds between the training and test sets. The kernel functions considered initially were linear, quadratic, RBF, and sigmoid, with the linear kernel yielding the best results in terms of R^2^, Mean Absolute Error (MAE), and Mean Squared Error (MSE) for both the training and test sets.

#### 4.3.4. Boosted Tree (BT) Model of ***CI***

The Boosted Tree regression model was generated in Statistica v. 13.3 using the same training and test datasets as those used to generate the MLR and SVR models. The set of independent variables used in the BT models was the same as that used in MLR, ANN, and SVR analyses. The BT regression model was validated using R^2^ for the training sets and RMSE_pred_ (Root Mean Square Error of Prediction) for the test sets.

#### 4.3.5. Applicability Domain (AD) in ***CI*** Models

The reference set of 84 compounds taken from Giaginis et al. [62] was split into a training set (n = 70) and a test set (n = 14) using the Kennard–Stone algorithm [78]. Principal component analysis (PCA) was performed on the training set, and the number of principal components was selected based on the scree plot and the cumulative explained variance. It was assumed that the PC’s selected for further processing should account for ca. 70% of the total variance [79]. Using this criterion, a five-component model (K = 5) was selected. Class membership for both reference and new compounds was assigned using the combination of Hotelling’s T^2^ and Q measures [61].

#### 4.3.6. SIMCA Model of Secretion into Breast Milk

SIMCA was used as a one-class classifier to model the distribution of compounds in the context of their secretion into breast milk. The model was built in R v.4.5.2 (https://www.r-project.org, accessed on 2 January 2026), using a subset of 160 compounds (training set), and validated on a subset of 42 compounds (test set). Both the training and test sets were selected by the Kennard–Stone algorithm [78] from the reference set of 202 compounds reported by Vijayaraghavan et al. [63]. Principal component analysis (PCA) was performed on the training set, and the number of principal components was selected using 5-fold cross-validation, with the false negative rate (FN) as the criterion. Using this criterion, a two-component model (K = 2) with zero FN in cross-validation was selected. Class membership was assigned using a combination of Hotelling’s T^2^ and Q measures [61].

#### 4.3.7. OC-PLS Model of Secretion into Breast Milk

A one-class partial least squares (OC-PLS) classifier was constructed using Python v. 3.13.13 (https://www.python.org), following the approach proposed by Xu et al. [72]. The model was built for the target class using the relationship 1 = Xb_PLS_ + e, where X is the descriptor matrix of the reference compounds, the response vector contains only “1”, and e represents the model residuals. The columns of X were not mean-centered. The 202 reference compounds were divided by the Kennard–Stone algorithm [78] into a training set (n = 160) and a test set (n = 42). The number of significant latent variables was determined by Monte Carlo cross-validation (MCCV) [80] using the minimum predicted residual sum of squares (PRESS) criterion [77]. The MCCV settings were selected according to [72]: 100 sampling runs and 20% of the objects left out in each iteration. The model’s parameters are presented in Table 2.

## 5. Conclusions

All studied cannabinoids might cross the placenta, as suggested by their predicted placental clearance index (***CI***) relative to antipyrine. The predicted ***CI*** values are particularly high (but still lower than those reported for antipyrine) for THC, THCV, CBD, CBDV, CBC, and CBG.

Cannabinoids might also be secreted into breast milk if consumed by a female during lactation. Based on the one-class models developed in this study, the cannabinoids least likely to enter human breast milk are bulky molecules such as cannabisol (Obs23), CBDD (Obs60), and CBDA-C5 9-OH-CBT-C5 (Obs97). Interestingly, these compounds do not have particularly low predicted ***CI*** values (which would imply severely restricted placental passage). However, two of these compounds (Obs23 and Obs60) fall outside the Applicability Domain of our ***CI*** predictive models, as determined by the Q vs. Hotelling’s T^2^ analysis presented above, so predictions of their ability to cross the placenta using the models developed in this study may be inaccurate.

## Figures and Tables

**Table 1:** Summary of predicted endpoints for sample cannabinoids selected according to [51].

		Transplacental Transport		Secretion into Milk		
No.	Compound	*CI_pred_*	Empirical	SIMCA	OC-PLS	Empirical
Obs1	THC	0.83	Yes	in-class	in-class	Yes
Obs2	THCAA	0.55		in-class	in-class	
Obs3	THCAB	0.66		in-class	in-class	
Obs6	THCV	0.82		in-class	in-class	
Obs31	CBG	0.78		in-class	in-class	
Obs51	CBD	0.82	Yes	in-class	in-class	Yes
Obs52	CBDA	0.58		in-class	in-class	
Obs55	CBDV	0.85		in-class	in-class	
Obs71	CBC	0.79		in-class	in-class	

**Table 2:** Key parameters of the OC-PLS model.

Metric	Value
Alpha	0.99
Selected LV (K)	4
mu_e	0.024297
sigma_e	0.160958
SD limit	14.11938
ACR limit	0.414601
Mean Predicted_y (Training)	0.920433
Mean Predicted_y (Test)	0.907495
Mean Predicted_y (Studied)	0.997848

## Data Availability

Data provided in the manuscript or Supplementary Materials. Further inquiries can be directed to the corresponding author.

## Supplementary Materials

The following supporting information can be downloaded at: https://www.mdpi.com/article/10.3390/ijms27146446/s1.
